# Identification and Evolution of Functional Alleles of the Previously Described Pollen Specific Myrosinase Pseudogene *AtTGG6* in *Arabidopsis thaliana*

**DOI:** 10.3390/ijms17020262

**Published:** 2016-02-22

**Authors:** Lili Fu, Bingying Han, Deguan Tan, Meng Wang, Mei Ding, Jiaming Zhang

**Affiliations:** Institute of Tropical Bioscience and Biotechnology, Key Laboratory of Tropical Crops Biology and Genetic Resources, Ministry of Agriculture, CATAS, Haikou 571101, China; fulili@itbb.org.cn (L.F.); hanbingying@itbb.org.cn (B.H.); tandeguan@itbb.org.cn (D.T.); wangmeng1981_wm@126.com (M.W.); bohemore@163.com (M.D.)

**Keywords:** myrosinase, β-thioglucosidase, *AtTGG6*, *AlTGG6*, evolution, pollen specific, frame-shift mutation

## Abstract

Myrosinases are β-thioglucoside glucohydrolases and serve as defense mechanisms against insect pests and pathogens by producing toxic compounds. *AtTGG6* in *Arabidopsis thaliana* was previously reported to be a myrosinase pseudogene but specifically expressed in pollen. However, we found that *AlTGG6*, an ortholog to *AtTGG6* in *A. lyrata* (an outcrossing relative of *A. thaliana*) was functional, suggesting that functional *AtTGG6* alleles may still exist in *A. thaliana*. *AtTGG6* alleles in 29 *A. thaliana* ecotypes were cloned and sequenced. Results indicate that ten alleles were functional and encoded Myr II type myrosinase of 512 amino acids, and myrosinase activity was confirmed by overexpressing *AtTGG6* in *Pichia pastoris*. However, the 19 other ecotypes had disabled alleles with highly polymorphic frame-shift mutations and diversified sequences. Thirteen frame-shift mutation types were identified, which occurred independently many times in the evolutionary history within a few thousand years. The functional allele was expressed specifically in pollen similar to the disabled alleles but at a higher expression level, suggesting its role in defense of pollen against insect pests such as pollen beetles. However, the defense function may have become less critical after *A. thaliana* evolved to self-fertilization, and thus resulted in loss of function in most ecotypes.

## 1. Introduction

Plant myrosinase (β-thioglucoside glucohydrolase, EC 3.2.1.147) belongs to one of the currently 135 families in the glycoside hydrolase superfamily [1,2]. It is a unique class of glycosidases by catalyzing the hydrolysis of S-linked glucosides, glucosinolates (anionic 1-thio-β-d-glucoside *N*-hydroxysulphates). Glucosinolates occur mainly in Brassicale plants [3], and release toxic compounds when hydrolyzed by myrosinase. Thus, myrosinase plays important roles in the defense system of cruciferous plants [4,5].

All crucifers analyzed so far have multiple forms of myrosinases. In oilseed rape (*Brassica napus*) and other *Brassica* species, approximately 25–30 myrosinase isoforms may be present [6,7,8]. These myrosinases were divided into three subgroups (MA, MB, and MC) based on their biochemical properties and sequence similarities [8]. *Arabidopsis thaliana* was shown to have six myrosinase genes *AtTGG1–6*, among which *AtTGG1* and *AtTGG2* were functional and expressed in above ground tissues [4,9], while *AtTGG4* and *AtTGG5* were expressed in roots [10,11]. Interestingly, *AtTGG3* and *AtTGG6* were disabled in tested ecotypes, but specifically expressed in anthers [12,13]. *Carica papaya* had at least three myrosinase genes [14,15], which were clustered together with *AtTGG4* and *AtTGG5* in a new subgroup of myrosinases Myr II, while MA, MB, and MC myrosinases together with *AtTGG1–3* were clustered in another subgroup Myr I by phylogenetic analysis [14,15]. Myr I and II were also different by their exon/intron organizations, in which Myr I had 12 exons and used an unusual intron splicing border GC/AG for intron 1 [16], while Myr II had 13 exons and used the unusual intron splicing border GC/AG for intron 10 and the normal border GT/AG for intron 1. However, papaya myrosinase genes did not use any unusual splicing border [14,15].

The anther specific expression of the disabled *AtTGG6* in *A. thaliana* suggested its possible function in the evolutionary history [12], and moreover, database searches in *Arabidopsis lyrata* genome identified a functional ortholog *AlTGG6*, which suggested that functional alleles of *AtTGG6* may still exist in *A. thaliana* ecotypes. In this study, we report identification of surprisingly high frequency of functional alleles and highly polymorphic frame-shift mutations in the non-functional *AtTGG6* alleles.

## 2. Results

### 2.1. Identification of Functional Alleles of the AtTGG6 Locus in Arabidipsis Thaliana

**Figure 1:**
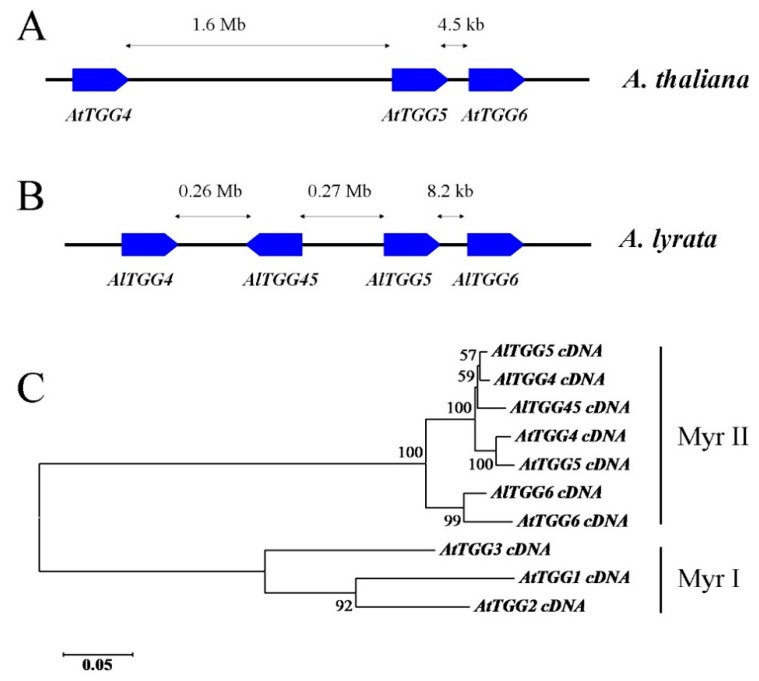
Chromosomal locations and phylogenetic analysis of Myr II members in *Arabidopsis lyrata* and *A. thaliana*. (**A**) Chromosomal locations of Myr II genes in *A. thaliana*; (**B**) Chromosomal locations of Myr II genes in *A. lyrata*; (**C**) Phylogeny inferred from cDNAs of Myr II members in *A. thaliana* and *A. lyrata*, using Myr I myrosinases *AtTGG1* (At5g26000), *AtTGG2* (At5g25980), and *AtTGG3* (At5g48375) as outgroups. Note: *AtTGG4* (At1g47600) and *AtTGG5* (At1g51470) cDNA sequences were from ecotype Col-0, *AtTGG6* (At1g51490) sequence was from ecotype Tsu-1 (GenBank accession number KU301834). *AlTGG* cDNA sequences were obtained by annotating a genomic scaffold (NW_003302555) in *A. lyrata*, and partially confirmed by sequencing the PCR-amplified cDNAs. The sequences were deposited in the GenBank database under accession numbers KU301856, KU301857, KU301858, and KU301859 for *AlTGG4*, *AlTGG5*, *AlTGG6*, and *AlTGG45*, respectively.

Myrosinase gene *AtTGG6* is a Myr II member gene in *Arabidopsis thaliana* and was previously reported to be disabled in five tested ecotypes [12]. Interestingly, it was predominantly expressed in pollen [12]. Blast searches in *Arabidopsis lyrata* subsp. *lyrata* genome resulted in four Myr II type myrosinase gene members designated here as *AlTGG4*, *AlTGG5*, *AlTGG6*, and *AlTGG45*. They locate in the chromosome with an organization similar to the Myr II genes *AtTGG4*, *AtTGG5*, and *AtTGG6* in *A. thaliana* (Figure 1A), except that *AlTGG4* and *AlTGG5* are separated by the inverted gene *AlTGG45* (Figure 1B). All the four *A. lyrata* myrosinase genes have the same exon/intron organizations as the Myr II members with 13 exons and the unusual intron splicing border GC/AG for intron 10, suggesting that this intron splicing border had existed before the two species were diverged. *AlTGG4*, *AlTGG5*, and *AlTGG6* encode preproteins of 510, 510, and 512 residues, respectively, and are likely functional, while *AlTGG45* is likely non-functional due to a 2412 bp insertion in exon 3 (GenBank accession number KU301859).

Phylogenetic analysis indicated that *AlTGG4*, *AlTGG5*, and *AlTGG45* were clustered together with *AtTGG4* and *AtTGG5* in the Myr II clade, but they were subdivided by species (Figure 1C), suggesting that *TGG4* and *TGG5* in the genus *Arabidopsis* were duplicated after the two species had diverged. However, *AtTGG6* and *AlTGG6* were clustered in another subgroup in the Myr II clade (Figure 1C), which suggested that *AlTGG6* is an ortholog to *AtTGG6*, and functional *AtTGG6* alleles may still exist in some ecotypes of *A. thaliana*.

*AtTGG6* genomic DNAs were amplified and sequenced in 29 *A. thaliana* ecotypes. In contrast to our expectation, as many as 10 ecotypes contained functional alleles. cDNAs of the functional alleles in two representative ecotypes Tsu-1 and Ty-0 were cloned and sequenced. They encoded preproteins of 512 residues in which seven residues were different between them, and they shared approximately 95% amino acid identities with AlTGG6. A secretion signal of 22 residues was identified by the SignalP 4.0 server [17], and the predicted molecular mass after signal excision was 55.7 kD, and *pI* was 9.4.

**Figure 2:**
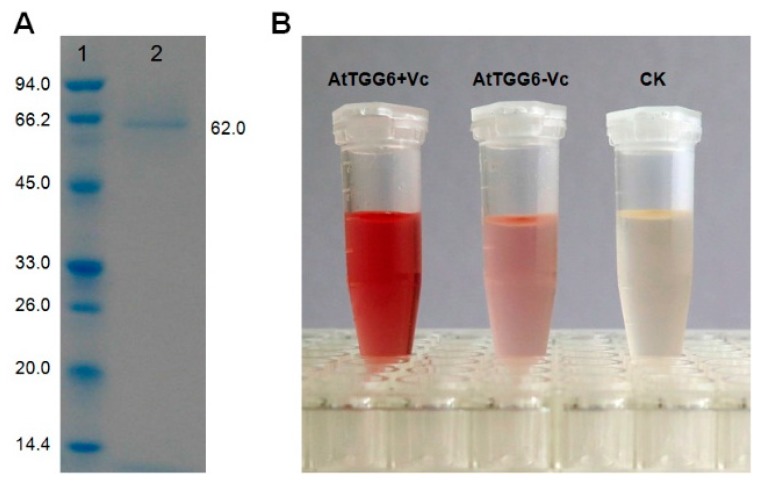
Myrosinase activity of recombinant AtTGG6 from *Arabidopsis* ecotype Tsu-1. (**A**) Sodium dodecyl sulfate-polyacrylamide gel electrophoresis (SDS-PAGE) of recombinant protein overexpressed in *Pichia pastoris* GS115; **Lane 1**, molecular weight marker, the mass of each band in kilodalton (kD) is shown to the left; **Lane 2**, purified recombinant AtTGG6; (**B**) Myrosinase activity of purified AtTGG6 as visualized using a glucose test reagent; AtTGG6 + Vc, AtTGG6 (50 ng) plus 0.8 mM ascorbic acid (Vc); AtTGG6 − Vc, AtTGG6 (50 ng) only, Vc not added; CK, AtTGG6 (50 ng) disabled by heating at 95 °C for 5 min before use.

The *AtTGG6* from ecotype Tsu-1 was overexpressed in *Pichia pastoris*. The purified recombinant protein had a molecular weight of approximately 62 kD (Figure 2), higher than the predicted mass, possibly due to glycosylation, which is common in myrosinase [14,15,18]. Myrosinase activity of the purified protein was confirmed by its ability to release glucose from sinigrin, a common myrosinase substrate (Figure 2). The production of glucose was visualized using a glucose test reagent containing glucose oxidase and 4-aminoantipyrin (GOD-PAP). Myrosinase activity of the recombinant AtTGG6 was activated by adding 0.8 mM ascorbic acid in the reaction mixture (Figure 2). Ascorbate (Vc) activation is a well-known plant myrosinase characteristic [15,19,20,21].

The exon/intron structure of the functional *AtTGG6* was the same as the Myr II members with 13 exons and the unusual GC/AG splicing border for intron 10. When the sequences of the functional alleles were aligned with the disabled allele in *A. thaliana* ecotype Col-0, all the frame-shift mutations in Col-0 previously predicted were confirmed [12] (Figure S1).

### 2.2. Highly Active Frame-Shift Mutations in AtTGG6 Alleles

The *AtTGG6* locus is a hot spot for frame-shift mutations. Among the 19 disabled alleles, a total of 13 frame-shift mutation types were identified, which were designated as *Mu1* to *Mu13* (Table 1). The mutations include insertion and deletion mutations (InDels) in exons, such as one base insertion in exon 3 (*Mu3*), exon 6 (*Mu4*), and exon 11 (*Mu11*); two base insertion in exon 1 (*Mu1*) and exon 7 (*Mu5*); one base deletion in exon 12 (*Mu13*); four base deletion in exon 9 (*Mu6*); 14 base deletion in exon 9 (*Mu7*); 17 base deletion in exon 11 (*Mu8*) and exon 12 (*Mu12*); and 208 base deletion from intron 2 to exon 4 (*Mu2*). Mutations in the exon/intron splicing border, such as 3′-splicing border mutation from AG to AT (*Mu9*) and/or to GG (*Mu10*) were also identified, which resulted in splicing failure for intron 10. The failure in splicing was confirmed by sequencing the cDNAs in ecotype Gre-0 that contained *Mu9* and Stw-0 that contained *Mu10* (cDNA sequences have been deposited in GenBank at accession numbers KU301844 and KU301851, respectively). The ecotypes and relevant frame-shift mutations are listed in Table 2.

The predicted nine base deletion in the intron 10/exon 11 junction in ecotype Col-0 in our previous report [12] was actually 17 bases (*Mu8*) according to current alignment with the functional alleles (Figure S1), which includes four bases in intron 10 and 13 bases in exon 11. Four ecotypes (Col-0, Col-4, Col-J, and Cvi-0) contained this mutation, which resulted in retaining of intron 10 in the mRNA in ecotype Col-0 and Col-4 as confirmed by sequencing their cDNAs (GenBank accession numbers KU301839 and KU301840).

*Mu1* had the highest frequency, and eight out of the 19 disabled alleles contained this mutation, while *Mu2*, *Mu7*, and *Mu11* were identified in only one allele (Table 2). The 3′ splicing border region of the unusual intron 10 is one of the hottest targets for disable mutation. Four types of mutations (*Mu8*, *Mu9*, *Mu10*, and *Mu11*) were found in this region with a total of 10 events in 10 distinct ecotypes. All these events resulted in failure of intron splicing as revealed by sequencing the cDNAs of representative ecotypes (Table 2). It is not known whether there is a relationship between the rare intron splicing border and the mutation rate.

### 2.3. Genetic Diversity and Evolutionary History of AtTGG6

*AtTGG6* was highly diverged according to the genetic diversity parameters calculated with DnaSP5 software [22]. The overall sequence identities of *AtTGG6* alleles were only 85.4%. The haplotype diversity was 0.973 with 109.5 InDel sites and 36.5 single nucleotide polymorphic (SNP) sites per kb DNA (Table 3).

The diversity of exons and introns was calculated separately. Their haplotype diversity was similar, but the nucleotide diversity of introns was significantly higher than that of exons, and there were more InDels and SNPs in the introns (Table 3). These results suggested that there had been a selection pressure for a functional *AtTGG6* in the evolutionary history. This hypothesis was also supported by the diversity parameters of the functional and the disabled alleles calculated separately. The haplotype diversity, the population mutation rate *θ*w, InDel sites, and SNP sites were all higher in the disabled alleles than those in the functional alleles, except that the nucleotide diversity per site in the functional alleles was higher than that in the disabled alleles (Table 3). The higher nucleotide diversity in the functional alleles may have resulted from the accumulation of synonymous mutations in the evolutionary history. The disabled alleles were evolved from the functional alleles, and thus had a shorter evolutionary history compared to the functional alleles, and had accumulated less synonymous mutations. However, frame-shift mutations were accumulated in the population of the disabled alleles due to the lack of selection pressure after mutation.

**Figure 3:**
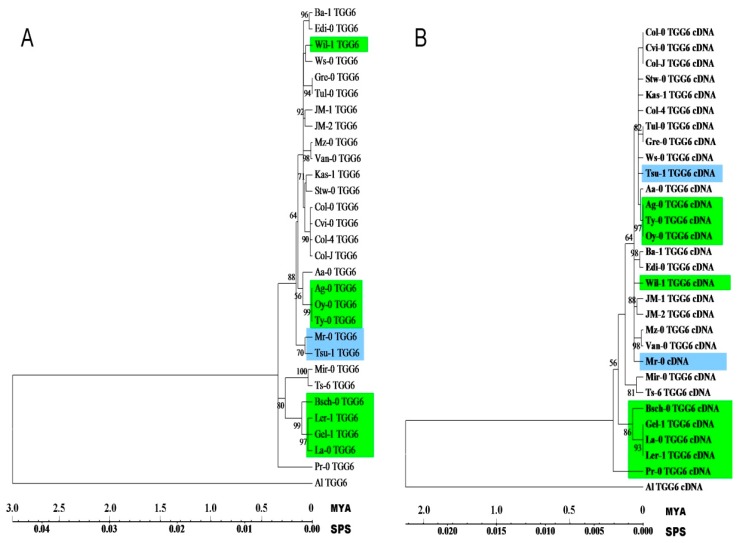
Phylogenetic analysis of *AtTGG6* genomic (**A**) and cDNA (**B**) sequences by Maximum Likelihood method. The evolutionary history was inferred based on the Tamura–Nei model [23]. The bootstrap values over 50% are shown next to the branches. Initial tree(s) for the heuristic search were obtained automatically. The tree is drawn to scale, with branch lengths measured in million years (MYA) and substitutions per site (SPS). Evolutionary analyses were conducted in MEGA6 [24]. Functional alleles are highlighted in green or blue. The two functional alleles in Tsu-1 and Mr-0 which are clustered different in genomic and cDNA phylogenies are highlighted in blue.

Phylogenetic analysis of *AtTGG6* genomic and cDNA sequences of the 29 alleles revealed that the disabled alleles dispersed in different clades represented by different functional alleles (Figure 3), suggesting that disable mutation events happened many times independently in the evolutionary history, and most of the events happened within 50 thousand years when genomic DNA was used to estimate the timing (Figure 3A), and the timing was narrowed down to a few thousand years when cDNA was used (Figure 3B), and this mutation rate may have been accelerated in human greenhouses where *A. thaliana* is grown for many generations a year.

### 2.4. Expression Pattern of the Functional Allele in Arabidopsis thaliana

**Figure 4:**
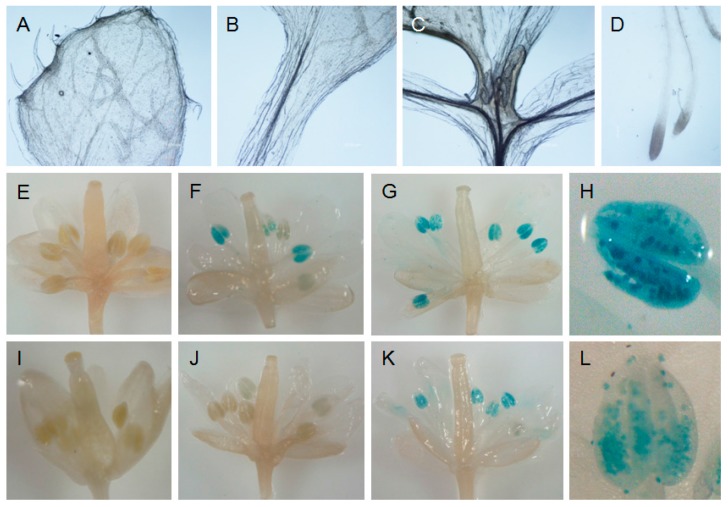
Expression pattern of functional (**A**–**H**) and disabled (**I**–**L**) alleles of *AtTGG6* in *Arabidopsis thaliana*. Functional *Prom::GUS* and disabled *Prom::GUS* were transformed into Col-0, and stained with X-gluxuronide solution. (**A**) Rosette; (**B**) stem; (**C**) leaves; and (**D**) root of transgenic plant with functional *Prom::GUS*; (**E**) a flower bud opened with a needle showing no *GUS* staining; (**F**) a flower one day before flowering opened with a needle showing the early expression; (**G**) a fully opened flower showing the high expression level; (**H**) an anther showing predominant expression in pollen; (**I**) a flower bud of disabled *Prom::GUS* opened with a needle showing no GUS staining; (**J**) a flower of disabled *Prom::GUS* one day before flowering showing weak expression; (**K**) a fully opened flower of disabled *Prom::GUS* showing expression at moderate level; (**L**) an anther showing disabled *AtTGG6* predominantly transcribed in pollen.

The promoters of the functional (from Tsu-1) and the disabled (from Col-0) *AtTGG6* were respectively fused with GUS gene and transformed into ecotype Col-0. GUS assay revealed that the functional *AtTGG6* was expressed predominantly in pollen, but not expressed in other tissues of the flower, neither expressed in leaves, flower stalk, and roots (Figure 4A–H). This expression pattern was similar to the disabled allele in Col-0 (Figure 4I–L). However, the expression level of the functional allele was higher than that of the disabled allele as visualized by darkness of blue color. These results indicated that the functional *AtTGG6* was possibly responsible for protection of pollen against pest insects such as pollen beetles, which caused severe crop loss in oilseed rape (*Brassica napus* L.) in some regions of the world [25].

## 3. Discussion

*AtTGG6* was previously suggested to be a pseudogene after sequencing the genes in five ecotypes [12]. Surprisingly, ten out of 29 ecotypes were found to have functional alleles in this study, and the expression pattern of the functional allele was similar to that of the disabled allele, which suggested that the functional *AtTGG6* was responsible for resistance against pollen herbivores in the evolutionary history based on known functions of myrosinases [8,26,27]. However, the necessity to defend pollen in *A. thaliana* may have become weaker than its outcrossing relative *A. lyrata*, after *A. thaliana* had become an autogamous species and can pollinate before the flower is open by disabling its self-incompatibility (SI) genes. SI genes still exist widely in crucifers [28,29,30]. Therefore, the strength or efficacy of natural selection for a functional *AtTGG6* decreased, and thus, disabled alleles survived, which may be one of the explanations for the high frequency of frame-shift mutations in *AtTGG6*. Gene expression patterns have been hypothesized not only to be the major determinants of nonsynonymous variation rate among genes, but also a critical determinant of gene retention [31,32]. A second factor that may have influenced the mutation of *AtTGG6* may be related to self-fertilization itself, which resulted in a large reduction in the effective rate of recombination and a corresponding decline in effective population size, and accelerated mutation [32,33]. A third factor that may have accelerated the mutation of *AtTGG6* may be the singleton nature of this gene in the genome. *AtTGG6* was localized closely to *AtTGG4* and *AtTGG5* in the chromosome (Figure 1A), however, phylogenetic analysis indicated that *AtTGG4* and *AtTGG5* were subdivided with *AlTGG4*, *AlTGG5*, and *AlTGG45* in *A. lyrata* by species in the Myr II clade (Figure 1C), suggesting that *TGG4* and *TGG5* in the genus *Arabidopsis* were duplicated recently, while *TGG6* was a singleton, more diverged, and was evolved before *A. thalina* and *A. lyrata* were separated into two species (Figure 1C). Singletons had a higher frequency of evolutionary rate than duplicated genes [31].

Frame-shift mutation was a major force to drive *AtTGG6* to lose function. We observed highly active frame-shift mutations in the *AtTGG6* locus. A total of 13 frame-shift mutation types were identified in 29 ecotypes (Table 2). These disabled alleles were dispersed in different clades in the phylogenetic tree (Figure 3), suggesting that *AtTGG6* was disabled many times independently in the evolutionary history. It is well known that myrosinase use GC/AG alternative intron splicing border [12,16]; Myr I and Myr II member genes use GC/AG for intron 1 and intron 10, respectively. Here, all the *AtTGG6* alleles were found to use GC/AG for intron 10, except for the specific splicing mutants. Interestingly, the unusual intron splicing border region in *AtTGG6* gene was a hot spot for frame-shift mutations (Table 2). A total of 10 frame-shift mutation events were identified in this region, including the mutations of splicing border from GC/AG to GC/AT and GC/GG. All these events resulted in failure of intron splicing as revealed by sequencing the cDNA of representative ecotypes. It is not known whether there is a relationship between the rare intron splicing border and the mutation frequency. Alternative splicing is common in plants [34,35]. However, alternative transcripts of *AtTGG6* were not obtained and the GC/AT and GC/GG splicing borders in *AtTGG6* were also proven not functional by RT-PCR analysis.

The functional alleles of *AtTGG6* are retained in some ecotypes possibly due to insufficient evolutionary time, since self-fertilization in *A. thaliana* occurred just a million years ago [30], after *A. thaliana* was diverged from its outcrossing relative *A. lyrata* approximately 5 million years ago [28,32], and from *Brassica species* 15 to 20 million years ago [36]. Through analysis of sequence diversity and phylogenetic analysis, frame-shift mutations of *AtTGG6* may have happened many times in the evolutionary history, and most mutations happened only a few thousand years ago (Figure 3), some may have even occurred recently in greenhouses.

## 4. Experimental Section

### 4.1. Plant Materials and Growth Conditions

Most ecotypes of *A. thaliana* are obtained from the *Arabidopsis* Biological Resource Center (ABRC) at The Ohio State University (Columbus, OH, USA). Some ecotypes are old stocks in our lab. Their accession numbers and origins are listed in Table 4. The plants were grown in a culture room under 16/8 h photoperiod, 200 μmol·m^−2^·s^−1^ light intensity, and 20 °C.

### 4.2. DNA Isolation and Cloning of AtTGG6 Genomic Genes

Leaves collected from the ecotypes were grounded in liquid nitrogen. DNA was isolated with a Universal Genomic DNA Extraction Kit Ver.3.0 (TaKaRa Biotechnologies, Dalian, China). Full length genomic gene of *AtTGG6* was amplified with gene specific primers G6F1 (5′ ACC CGC TGA AAA GCT CCA TCA A 3′) and G6R1 (5′ GGC TTC CAC TTA TTT TGC AAT GAA CC 3′) and sequenced at Shanghai ShineGene Molecular Biotechnology Co., Ltd., Shanghai, China.

### 4.3. Isolation of mRNA, RT-PCR and Sequence Analysis

Plant samples of representative ecotypes were grounded in liquid nitrogen. Total RNA was isolated with 3S Trizol Total RNA isolation reagents (Shenergy Biocolor Bioscience & Technology Company, Shanghai, China). mRNA was then purified from the total RNA with an Oligotex Direct mRNA purification kit (Qiagen GmbH, Hilden, Germany). Moloney Murine Leukemia Virus (M-MuLV) reverse transcriptase (TaKaRa Biotechnologies, Dalian, China) was used to generate first strand cDNA. cDNA fragments of *AtTGG6* were amplified with gene specific primers G6F1 and G6R1 and cloned in pMD19-T vector (TaKaRa Biotechnologies, Dalian, China). Both the PCR fragments and the clones were sequenced at ShineGene, Shanghai. The sequences were analyzed with MacVector software (Version 13.5.5, Oxford Molecular, Oxford, UK).

### 4.4. Overexpression of AtTGG6 in Pichia Pastoris

A full length cDNA clone containing the correct *AtTGG6* sequence of ecotype Tsu-1 was selected to construct an intracellular expression vector. The coding region was re-amplified with primers HNP38 (5′ GGG TGA TCA GCA TGG CAA TTC CAA AAG CTC ACT ACT CT 3′) and HNP39 (5′ GGG CCT AGG CTA GTG GTG ATG GTG ATG GTG TTT TGC AAT GAA CCT AGA GAA CCA TTT TCC A 3′), using proofreading DNA polymerase LA Taq (TaKaRa Biotechnologies). The PCR reaction was performed for 30 cycles, at 94 °C for 30 s, 60 °C for 30 s, 72 °C for 3 min and 30 s. The product was digested with *Bcl* I and *Avr* II, and ligated into an intracellular expression vector *pPIC3.5* (Invitrogen, San Diego, CA, USA) that was previously digested with *Bam* HI and *Avr* II. The ligated product was transformed into *Escherichia coli* by electroporation. The recombinant plasmid with correct sequence was chosen to transform *P. pastoris* strain GS115 (Invitrogen) as previously described [14].

### 4.5. Purification of Recombinant AtTGG6 and Analysis of Myrosinase Activity

A single *P. pastoris* colony with high myrosinase activity was used to initiate large scale cultures as described previously [14]. The production of recombinant myrosinase was induced by addition of 0.5% methanol each day for five days. The his-tagged recombinant protein was isolated and purified with an 1-mL BioRad profinity cartridge as described previously [14]. The purity and molecular weight of recombinant enzyme was examined with a 4%–10% Bis–Tris SDS-PAGE gel and stained with Coomassie brilliant blue. Myrosinase activity was tested by its ability to release glucose from commonly used substrate sinigrin (Sigma Chemical Co., St. Louis, MO, USA). Glucose produced in the reaction was visualized by reaction with a glucose test reagent (Shanghai Rongsheng Biotech Co., Ltd., Shanghai, China) that is widely used to measure blood glucose level in hospitals.

### 4.6. Construction of AtTGG6 Prom::GUS Expression Vector, Transformation, and GUS Staining

The promoter of *AtTGG6* was amplified as previously described with primers HNP56 (5′ GGA CTT TGG ATC ACC ATT AAG CTT GCA G 3′) and HNP57 (5′ GTT GGA TCC GCT TTC TTT TTG CGT TTC TTG ATG GAG 3′) [12] from ecotype Tsu-1 and Col-0. The product was digested with *Hind* III and *Bam* HI, and inserted into the *pBI121* vector pre-digested with *Hind* III and *Bam* HI. The recombinant plasmids containing *AtTGG6 Prom::GUS* were transformed into *E. coli* and screened by restriction digestion and sequencing. The correct expression vectors were transformed into *Agrobacterium tumefaciens* C58 by electroporation, and were further transformed into *Arabidopsis* ecotype Col-0 using vacuum infiltration [37]. Transformants were screened on MS medium containing 40 mg·L^−1^ kanamycin. The expression of *GUS* gene was detected as described [38].

### 4.7. Analysis of DNA Sequences and Genetic Diversity

Sequences were assembled using the Assembler program incorporated in the MacVector 13.5.5 software (MacVector, Inc.). The sequences of *AtTGG6* alleles were deposited in GenBank under accession numbers (KU301827–KU301855; Table S1). The Myr II member genes in *A. lyrata* were submitted in GenBank with accession numbers KU301856–KU301859 (Table S1). The exon/intron organizations and the frame-shift mutations in the *AtTGG6* alleles in different ecotypes were identified by aligning the genomic DNA sequences with the cDNA in ecotype Tsu-1 using MacVector software. Genetic diversity was analyzed as previously described [39]. Genomic sequences of the 29 alleles were aligned using ClustalX 2.0 [40]. The alignment results were exported into DnaSP 5.10.01 software [22]. The haplotype diversity, the nucleotide diversity, and the population mutation rate (Watterson estimator *θ*w [41]) were estimated using the default parameters of the software in all cases [22].

To analyze the diversity differences of exons and introns, the exon and intron sequences were extracted from genomic sequences and analyzed as described above. The significance of the differences was tested with one-way ANOVA at 5% confidence level.

## Figures and Tables

**Table 1:** Frame-shift mutations identified in *AtTGG6* alleles in *Arabidopsis thaliana* ecotypes.

Mutation	Location
*Mu1*	Two base insertion at +155 * in exon 1
*Mu2*	208 base deletion from +437 in intron 2 to +644 in exon 4
*Mu3*	one-base insertion at +481 in exon 3
*Mu4*	one-base insertion at +1007 in exon 6
*Mu5*	Two-base insertion at +1158 in exon 7
*Mu6*	four-base deletion from +1739 to +1742 in exon 9
*Mu7*	14 base deletion from +1804 to +1817 in exon 9
*Mu8*	17 base deletion from +2111 in intron 10 to +2127 in exon 11
*Mu9*	3’ splicing border mutation from AG to AT for intron 10
*Mu10*	3’ splicing border mutation from AG to GG for intron 10
*Mu11*	One-base insertion at +2121 in exon 11 close to 3’ splicing border of intron 10
*Mu12*	17 base deletion from +2311 to +2327 in exon 12
*Mu13*	One nucleotide deletion at +2348 in exon 12, 20 nucleotides after *Mu12*

* The number was counted from the first nucleotide of the start codon in *AtTGG6* gene of ecotype Tsu-1 (GenBank accession number KU301834).

**Table 2:** Distribution of frame-shift mutations in *AtTGG6* alleles among *Arabidopsis* ecotypes.

Ecotype	*Mu1*	*Mu2*	*Mu3*	*Mu4*	*Mu5*	*Mu6*	*Mu7*	*Mu8*	*Mu9*	*Mu10*	*Mu11*	*Mu12*	*Mu13*	Functionality
Aa-0	+	−	−	−	−	−	−	−	−	−	−	−	−	disabled
Ag-0	−	−	−	−	−	−	−	−	−	−	−	−	−	functional
Ba-1	−	−	−	−	−	−	−	−	−	−	−	−	+	disabled
Col-0	+	−	−	+	−	+	−	+ *	−	−	−	+	−	disabled
Col-4	+	−	−	+	−	+	−	+ *	−	−	−	+	−	disabled
Col-J	+	−	−	+	−	+	−	+	−	−	−	+	−	disabled
Cvi-0	+	−	−	+	−	+	−	+	−	−	−	+	−	disabled
Bsch-0	−	−	−	−	−	−	−	−	−	−	−	−	−	functional
Edi-0	−	−	−	−	−	−	−	−	−	−	−	−	+	disabled
Gel-0	−	−	−	−	−	−	−	−	−	−	−	−	−	functional
Gre-0	−	−	+	−	−	−	−	−	+ *	−	−	−	−	disabled
JM-1	−	−	−	+	−	−	−	−	−	−	−	−	−	disabled
JM-2	−	−	−	+	−	−	−	−	−	−	−	−	−	disabled
Kas-1	+	−	−	−	−	−	−	−	−	−	−	−	−	disabled
La-0	−	−	−	−	−	−	−	−	−	−	−	−	−	functional
Ler-1	−	−	−	−	−	−	−	−	−	−	−	−	−	functional
Mir-0	−	−	−	−	+	−	−	−	−	−	+ *	−	−	disabled
Mz-0	−	−	−	−	−	−	−	−	−	+	−	−	−	disabled
Mr-0	−	−	−	−	−	−	−	−	−	−	−	−	−	functional
Oy-0	−	−	−	−	−	−	−	−	−	−	−	−	−	functional
Pr-0	+	+	N/A **	−	−	−	+	−	−	−	−	−	−	disabled
Stw-0	+	−	−	−	−	−	−	−	−	+ *	−	−	−	disabled
Ts-6	−	−	−	−	+	−	−	−	−	−	−	−	−	disabled
Tsu-0	−	−	−	−	−	−	−	−	−	−	−	−	−	functional
Tul-0	−	−	+	−	−	−	−	−	+	−	−	−	−	disabled
Ty-0	−	−	−	−	−	−	−	−	−	−	−	−	−	functional
Van	−	−	−	−	−	−	−	−	−	+	−	−	−	disabled
Ws-0	−	−	+	−	−	−	−	−	−	−	−	−	−	disabled
Wil-0	−	−	−	−	−	−	−	−	−	−	−	−	−	functional
Frequency	8	1	3	6	2	4	1	4	2	3	1	4	2	-

* Confirmed by sequencing cDNA sequences amplified with RT-PCR method; ** Not applicable, since *Mu2* in ecotype Pr-0 is a large deletion that spans the *Mu3* region.

**Table 3:** Genetic diversity in *AtTGG6* alleles in 29 *Arabidopsis thaliana* ecotypes.

Diversity Parameters	Genomic Gene	Exons	Introns	Functional Alleles	Nonfunctional Alleles
Sequence identities	85.4%	86.7%	80.5%	97.5%	86.0%
Haplotype diversity	0.973 ± 0.018	0.968 ± 0.019	0.948 ± 0.024	0.911 ± 0.077	0.959 ± 0.036
Nucleotide diversity per site	0.00758 ± 0.00085	0.00593 ± 0.00055	0.01051 ± 0.00167	0.00770 ± 0.00082	0.00666 ± 0.00140
*θ*w per site	0.01043 ± 0.00336	0.00968 ± 0.00323	0.01199 ± 0.00405	0.00691 ± 0.00291	0.01027 ± 0.00364
SNP sites per kb	36.485	34.282	39.792	5.605	32.030
InDel sites per kb	109.456	98.318	154.844	19.432	107.635
Total aligned length	2686	1546	1156	2676	2685

**Table 4:** Ecotypes used in this research.

Ecotypes	ABRC Stock Number	Location
Aa-0	CS937	Aua/Rhon, Germany
Ag-0	CS936	Argentat ,France
Ba-1	CS952	Blackmount, United Kingdom
Bsch-0	CS1002	Buchschlag/Frankfurt am Main, Germany
Col-0	CS6673	Columbia, SC, USA
Col-4	CS933	Columbia, SC, USA
Cvi-0	CS1096	Cape Verde Islands, Cape Verde
Edi-0	CS1122	Edinburgh, United Kingdom
Gel-1	CS28279	Geleen, Netherland
Gre-0	CS1210	Greenville, MI, USA
Kas-1	CS903	Kashmir, India
La-0	CS1298	Landsberg/Warthe, Germany
Ler-1	CS1642	Landsberg, Germany
Mir-0	CS1378	Miramare/Trieste, Italy
Mr-0	CS1372	Monte/Tosso, Italy
Mz-0	CS1382	Merzhausen/Ts, Germany
Oy-0	CS1436	Oystese, Norway
Pr-0	CS1474	Frankfurt-Praunheim, Germany
Stw-0	CS1538	Stobowa/Orel, Russia
Tu1-0	CS1570	Turk Lake, MI, USA
Ts-6	CS1561	Tossa del Mar, Spain
Tsu-1	CS1640	Tsushima, Japan
Ty-0	CS1572	Taynuilt, United Kindom
Van-0	CS1584	Vancuuver, Canada
Wil-1	CS1594	Wilna/Litauen, Russia
Ws-0	CS1602	Wassilewskija, Russia
Col-J	-	Derived from Col-0
JM-1	-	Lab stock, unknown origin
JM-2	-	Lab stock, unkown origin

ABRC: Arabidopsis Biological Resource Center.

## Supplementary Materials

Supplementary materials can be found at http://www.mdpi.com/1422-0067/17/2/262/s1.
