# Supplementary material for: Identification and Evolution of Functional Alleles of the Previously Described Pollen Specific Myrosinase Pseudogene AtTGG6 in Arabidopsis thaliana
Source: Int J Mol Sci. 2016 Feb 22;17(2):262. doi: 10.3390/ijms17020262 (PMC4783991; doi:10.3390/ijms17020262)
Supplement: Supplementary file 1 [file ijms-17-00262-s001.pdf]

Supplementary Materials: Identification and Evolution of Functional Alleles of the Previously Described Pollen Specific Myrosinase Pseudogene *AtTGG6* in *Arabidopsis thaliana*

Lili Fu, Bingying Han, Deguan Tan, Meng Wang, Mei Ding and Jiaming Zhang

```
   1 ATGGCAATTCCAAAAGCTCACTACTCTTTAGCCATCATTGTCGTTCTTTTTGCCGTTTCGAGGAGCCAAAAAGTATGCAATCCAGAATGCAAGGCCAAAG Tsu
   1 ATGGCAATTCCAAAAGCTCACTACTCTTTAGCCATCATTGTCGTTCTTTTTGCCGTTTCGAGGAGCCAAAAAGTATGCAATCCAGAATGCAAGGCCAAAG Col

 101 AACCCTTCAACTGCGACAAAACTCTTGCATTCAACCGAAATGGATTTCCCAACAA--TTTTACTTTTGGTGCAGCTACCTCTGCATACCAGgtttacata  Tsu
 101 AACCCTTCAACTGCGACAAAACTCTTGCATTCAACCGAAATGGATTTCCCAAAAAAATTTTACTTTTGGTGCAGCTACCTCTGCATACCAGgtttacata  Col

 199 tatcttcgccatgatagctttaggggattgatttctagctataacattttccatatactttcttccttacaatatccaacgctttgttctatcagGTTGA  Tsu
 201 tatcttcgccatgatagatttaggggattgatttctagctataacattttccatatactttcttccttacaatatccaacgctttgttctatcagGTTGA  Col

 299 AGGTGCAGCACATAGAGCACTTAATGGATGGGACTATTTCACTCATAGATATCCAGgtcataaacatcatttaatattattatttattaacatgcatctt  Tsu
 301 AGGTGCAGCACATAGAGCACTTAATGGATGGGACTATTTCACTCATAGATATCCAGgtcataaacatcatttaatattattatttatta-catgcatctt  Col

 399 catgtatgaaaaagtataaatataagaaatatatattcatattcatatctcggattgttatacagAAAGAGTTTCAGATCGCAGTATAGGAGACCTTGCT  Tsu
 400 catgtatgtaaaagtataaatataagaaatatatattcatattcatatctcggattgttatacagAAAGAGTTTCAGATCGCAGTATAGGAGACCTTGCT  Col

 499 TGTAATTCGTATGATCTTTATAAGgtgaaaggctatcaatgacgaatgtgtacatatggtgtttgttcaatttttttcttagttttaatttaatttaaaga  Tsu
 500 TGTAATTCGTATGATCTTTATAAGgtgaaaggctatcaatgacgaatgtgtacatatggtgtttgttcaatttttttcttagttttaatttaatttaaaga  Col

 599 tttttcatataaccttgaacagGATGATGTTAAACTACTAAAAAGAATGAACGTTCAAGCATACCGATTCTCAATAGCATGGTCAAGGGTCTTACCAAgt  Tsu
 600 tttttcatataaccttgaacagGATGATGTTAAACTACTAAAAAGAATGAACGTTCAAGCATACCGATTCTCAATAGCATGGTCAAGGGTCTTACCAAgt  Col

 699 aaatggatcttcatgttttcatctaattataaatagaattcgtaaaattttacattccatcactaaactttaatttgtgattaaatgctaaatttagAGG  Tsu
 700 aaatggatcttcatgttttcatctaattataaatagaattcgtaaaattttacattccatcactaaactttaatttgtgattaaatgctaaatttagAGG  Col

 799 GCAGACTAATTGGAGGAGTCGACGAGAATGGGATCACATACTACAACAATCTCATTAACGAGTTGAAAGCAAATGgtaaattctaaattcaagtgcttaa  Tsu
 800 GCAGACTAATTGGAGGAGTCGACGAGAATGGGATCACATACTACAACAATCTCATTAACGAGTTGAAAGCAAATGgtaaattctaaattcaagtgcttaa  Col

 899 ttaatcatattttaaatcgttttagtatgtttgtgatatataaacatttaacttacatgaatacagGCATAGAACCATTTGTGACTATAATTCATTGGGA  Tsu
 900 ttaatcatattttaaatcgttttagtatgtttgtgatatataaacatttaacttacatgaatacagGCATAGAACCATTTGTGACTATATTTCATTGGGA  Col

 999 TGTTCCTCA-GACTTTAGAAGACGAATATGGAGGCTTCTTAAGCCCACGTATAGTgtaagtgtgttctatattctcttctttagcttagatatatgtaac  Tsu
1000 TGTTCCTCAAGACTTTAGAAGACGAATATGGAGGCTTCTTAAGCCCACGTATAGTgtaagtgtgttctatattctcttctttagcttagatatatgtaac  Col

1098 atcgcatcagatatgtgtctgtctaatatgtatgatacataaatagGGAGGACTTCAAAAATTACGCTGAGCTTCTATTCCAAAGATTCGGAGACAGAGT  Tsu
1100 atcgcatcagatatgtgtctgtctaatatgtatgatacataaatagGGAGGACTTCAAAAATTACGCTGAGCTTCTATTCCAAAGATTCGGAGACAGAGT  Col

1198 TAAATTTTGGATCACATTAAATCAGCCGTACTCTCTCGCTGTCAAAGGTTATGGAGATGGACAGTATCCACCGGGAAGGTGCACTGACTGTGAATTTGGA  Tsu
1200 TAAATTTTGGATCACATTAAATCAGCCGTACTCTCTCGCTGTCAAAGGTTATGGAGATGGACAGTATCCACCGGGAAGGTGCACTGACTGTGAATTTGGA  Col

1298 GGAGATTCTGGAACTGAACCTTATATAGTTGGACATCACGAACTTCTGGCTCATATGGAAGCTGTATCTTTATACCGAAAAAGATATCAGgtatacatac  Tsu
1300 GGAGATTCTGGAACTGAACCTTATATAGTTGGACATCACGAACTTCTGGCTCATATGGAAGCTGTATCTTTATACCGAAAAAGATATCAGgtatacatac  Col

1398 cgtaaactcataatttctctttgcgtcgatactactttgtggtatatttgagtctataatcaagaactacttacgcagAAATTTCAAGGTGGTAAGATAG  Tsu
1400 cgtaaactcataatttctctttgcgtcgatactactttgtggtatatttgagtctataatcaagaactacttacgcagAAATTTCAAGGTGGTAAGATAG  Col

1498 GAACGACATTGATCGGTAGATGGTTCATCCCACTAAATGAAACTAACGATCTCGACAAGGCTGCTGCAAAACGAGAATTCGATTTTTCCGTTGGCTGgta  Tsu
1500 GAACGACATTGATCGGTAGATGGTTCATCCCACTAAATGAAACTAACGATCTCGACAAGGCTGCTGCAAAACGAGAATTCGATTTTTCCGTTGGCTGgta  Col

1598 tatatgttcaactgatggattac-aaaacatt----atgtatatataaaaacatgctttgcaatattaatt-atgtatattttcttacatatgtgaagGT  Tsu
1600 tatatgttcaactcatggattaccaaaacattaattatgtatatataaaa-catgctgtgcaatattaatttatgtatattttcttacatatgtgtagGT  Col

1692 TCTTGGATCCACTGGTGTACGGACAATATCCAAAGATAATGAGAGACATGCTAGGAGATAGATTGCCAAAATTCACGCCCGAGCAATCAGCTTTACTTAA  Tsu
1699 TCTTGGATCCACTGGTGTACGGACAATATCCAAAGATAATGAGAGAC----TAGGAGATAGATTGCCAAAATTCACGCCCAAGCAATCAGCTTTACTTAA  Col

1792 AGGATCACTTGATTTTCTAGGGTTGAACTATTACGTTACAAGATATGCAACCTACAGACCTCCTCCAATGCCGACACAACATAGTGTCTTAACCGATTCA  Tsu
1795 AGGATCACTTGATTTTCTAGGGTTGAACTATTACGTTACAAGATATGCAACCTACAGACCTCCTCCAATGCCGACACAACATAGTGTCTTAACCGATTCA  Col

1892 GGAGTTACAATTGGATgtatgtcaaacatttgtcgtatttaggtggattttttcatatataaatcaccaaaactaatctttttataattggtttcagTTGA  Tsu
1895 GGAGTTACAATTGGATgtatgtcaaacatttgtcgtatttaggtggattttttcatatataaatcaccaaaactaatctttttataattggtttcagTTGA  Col

1992 GCGAAATGGAGTTTCTATTGGTGTTAAGgctagtatcgactttggtttcgtatattttttttcccagatgtgaaagatttacgacatttagttgattttt  Tsu
1995 GCGAAATGGAGTTTCTATTGGTGTTAAGgctagtatcaactttggtttcgtatattttttttcccagatgtgaaagatttacgacatttagttgattttt  Col

2092 ttttatttgtcgagcttctacagGCTCCAAGCTTCTCCTACTATCCACCAGGATTCCGTCAGATTCTAAATCACATCAAAAACAAGTACAAGAATCCATT  Tsu
2095 ttttatttgtcg----------------AGCTTCTCCTACTATCCACCAGGATTCCGTCAGATTCTAAATCACATCAAAAACAAGTACAAGAATCCATT  Col

2192 AACCTACATCACCGAAAACGgtatatgttgcattcaaatgtttaactttaaccaatgaaaaaactatttgtgttagttacaaatcttatgtcaatttgt  Tsu
2178 AACCTACATCACCGAAAACGgtatatgttgcattcaaatgtttaactttaaccaatgaaaaaactatttgtgttagttacaaatcttatgtcaatttgt  Col

2292 agGAGTTGCTGATGCTGATTTTGGAAACGTAACGATCGCAAATGCTCTTGCCGATAATGGACGAATTCAATTTCAATGCAGCCATCTTTCTTGTCTCAAA  Tsu
2278 agGAGTTGCTGATGCTGAT----------------CGCAAATGCTCTTGCCGATAATGGACGAATTCAATTTCAATGCAGCCATCTTTCTTGTCTCAAA  Col

2392 TGCGCAATTGAgtgagtttaatatatcattcgtatattatgtgatttactgtcttcggaga-ttatatatttaaattcat--caaaactatattttaaaa  Tsu
2361 TGCGCAATTGAgtgagtttaatatatcattcgtattttatgtgatttactgtcttcggagaattataaatttatattcatatcaaaactatttcttaaa  Col

2489 tatttaatagGGATGGATGCAACGTAGCAGGATATTTTGCGTGGTCATTGATGGACAATTATGAATTCGGAAATGGTTACACTCTCCGGTTTGGTATGAA  Tsu
2461 tatttaatagGGATGGATGCAACGTAGCAGGATATTTTGCATGGTCATTGATGGACAATTATGAATTCGGAAATGGTTACACTCTCCGGTTTGATATGAA  Col

2589 TTGGGTCAACTTCACTAATCCTGCTGATCGGAGAGAAAAAGCTTCTGGAAAATGGTTCTCTAGGTTCATTGCAAAATAA            2667     Tsu
2561 TTGGGTCAACTTCACTAATCCTGCTGATCGAAGAGAAAAAGCTTCTGGAAAATGGTTCTCTAGGTTCATTGCAAAATAA            2639     Col
```

Figure S1. Sequence comparison between the functional (from Tsu-1) and the disabled allele (from Col-0) of *AtTGG6* gene in *Arabidopsis thaliana*. The InDels leading to frame-shift mutations in Col-0 are highlighted in yellow. The exons are shown in capital letters, and the introns are shown in lower case.

Table S1. *AtTGG6* alleles from different ecotypes and their accession numbers in GenBank.

Allele	Accession Number
AtTGG6_genomic-fll.sqn AtTGG6_Ag-0	KU301827
AtTGG6_genomic-fll.sqn AtTGG6_Bsch-0	KU301828
AtTGG6_genomic-fll.sqn AtTGG6_Gel-1	KU301829
AtTGG6_genomic-fll.sqn AtTGG6_La-0	KU301830
AtTGG6_genomic-fll.sqn AtTGG6_Ler-1	KU301831
AtTGG6_genomic-fll.sqn AtTGG6_Mr-0	KU301832
AtTGG6_genomic-fll.sqn AtTGG6_Oy-0	KU30183a3
AtTGG6_genomic-fll.sqn AtTGG6_Tsu-1	KU301834
AtTGG6_genomic-fll.sqn AtTGG6_Ty-0	KU301835
AtTGG6_genomic-fll.sqn AtTGG6_Wil-1	KU301836
AtTGG6_genomic-fll.sqn AtTGG6_Aa-0	KU301837
AtTGG6_genomic-fll.sqn AtTGG6_Ba-1	KU301838
AtTGG6_genomic-fll.sqn AtTGG6_Col-0	KU301839
AtTGG6_genomic-fll.sqn AtTGG6_Col-4	KU301840
AtTGG6_genomic-fll.sqn AtTGG6_Col-J	KU301841
AtTGG6_genomic-fll.sqn AtTGG6_Cvi-0	KU301842
AtTGG6_genomic-fll.sqn AtTGG6_Edi-0	KU301843
AtTGG6_genomic-fll.sqn AtTGG6_Gre-0	KU301844
AtTGG6_genomic-fll.sqn AtTGG6_JM-1	KU301845
AtTGG6_genomic-fll.sqn AtTGG6_JM-2	KU301846
AtTGG6_genomic-fll.sqn AtTGG6_Kas-1	KU301847
AtTGG6_genomic-fll.sqn AtTGG6_Mir-0	KU301848
AtTGG6_genomic-fll.sqn AtTGG6_Mz-0	KU301849
AtTGG6_genomic-fll.sqn AtTGG6_Pr-0	KU301850
AtTGG6_genomic-fll.sqn AtTGG6_Stw-0	KU301851
AtTGG6_genomic-fll.sqn AtTGG6_Ts-6	KU301852
AtTGG6_genomic-fll.sqn AtTGG6_Tul-0	KU301853
AtTGG6_genomic-fll.sqn AtTGG6_Van-0	KU301854
AtTGG6_genomic-fll.sqn AtTGG6_Ws-0	KU301855
AlTGG4.sqn AlTGG4	KU301856
AlTGG4.sqn AlTGG5	KU301857
AlTGG4.sqn AlTGG6	KU301858
AlTGG4.sqn AlTGG45	KU301859
